# A large-scale dataset of functional mouse ganglion cell layer responses

**DOI:** 10.1038/s41597-026-08026-0

**Published:** 2026-08-11

**Authors:** Dominic Gonschorek, Jonathan Oesterle, Thomas Zenkel, Federico D’Agostino, Chenchen Cai, Nadine Dyszkant, Klaudia P. Szatko, Florentyna Deja, Tom Schwerd-Kleine, Ryan Arlinghaus, Katrin Franke, Zhijian Zhao, Timm Schubert, Philipp Berens, Thomas Euler

**Affiliations:** 1 Institute of Ophthalmic Research, University of Tübingen, Tübingen, Germany; 2 Werner Reichardt Centre for Integrative Neuroscience, University of Tübingen, Tübingen, Germany; 3 Hertie Institute for AI in Brain Health, University of Tübingen, Tübingen, Germany; 4 Tübingen AI Center, University of Tübingen, Tübingen, Germany; 5 Stanford University School of Medicine, Stanford, California USA; 6 Stanford Bio-X, Stanford, California USA; 7 Wu Tsai Neurosciences Institute, Stanford, California USA

## Abstract

We present the ALL-GCL dataset, a large-scale resource of functional two-photon Ca^2+^-imaging recordings with rich metadata information from more than 80,000 cells in the ganglion cell layer (GCL) of the ex vivo mouse retina. Collected over nine years across more than 155 experimental sessions, the dataset provides recordings of light-evoked responses to various stimuli, ranging from a shared set of core stimuli to natural movies. To enable cell-type-specific analyses, cells are probabilistically assigned to 46 previously characterised functional groups, including retinal ganglion cells and displaced amacrine cells. Further, we assessed the influence of experimental and biological factors on the functional responses. Classifier-based analyses identified measurable signatures associated with acquisition conditions and recording sessions, providing a quantitative characterisation of dataset structure and potential sources of batch effects. The ALL-GCL dataset offers a comprehensive and standardised reference for studying retinal computation at scale. It supports population-level analyses, computational modelling, and the development of machine learning approaches for biological time-series data. Future releases will expand the dataset with additional mouse lines and light stimuli, creating a growing resource for the vision science community.

## Background & Summary

Sensory systems are essential for survival, enabling organisms to perceive, interpret, and respond to their environment. Among mammals, vision is often an important if not the dominant sense, supporting critical behaviours such as navigation, foraging, and social interaction. Visual perception begins when photons reach the retina – a thin, multi-layered neuronal tissue at the back of the eye. Photoreceptors within the retina detect light and transduce it into electrical signals, which are relayed through bipolar cells (BCs) to retinal ganglion cells (RGCs), the output neurons of the retina to the rest of the brain^1,2^.

The retina’s primary task is to transform the continuous flow of light patterns from the environment into parallel representations of visual features such as intensity, contrast, “colour”, and motion^3^. This transformation is mediated by its highly organised circuit architecture, which integrates feed-forward and lateral interactions across multiple cell classes. Amacrine cells (ACs), for instance, modulate temporal and spatial aspects of RGC responses, shaping computations such as motion detection and contrast adaptation before signals are transmitted to the brain^4,5^. Through this sophisticated processing, the retina operates not merely as a camera sensor but as a dynamic computational network that actively extracts essential, behaviourally relevant features from the visual world^6^. Here, RGCs represent the retina’s output channels, encoding specific visual features and transmitting them to distinct downstream brain regions.

In the mouse retina, up to 45 functional RGC types have been identified^7,8^, a diversity mirrored by morphological and molecular classifications^9,10^. Each RGC type contributes to diverse aspects of visual encoding, ranging from edge detection and direction selectivity to luminance and chromatic contrast, also underscoring the retina’s capacity for distributed computation. Understanding how these diverse cell types collectively represent visual information remains a central question in vision research.

Progress in addressing this question increasingly depends on access to large, standardised datasets that capture the retina’s activity. While there is a plethora of studies that have provided valuable and detailed insights into individual RGC types and their local circuits, large-scale datasets that combine broad sampling with consistent experimental conditions and well-defined stimuli are still rare, or not easily publicly accessible. Yet, such resources are crucial for uncovering, for instance, response variability within RGC types, population-level principles, validating computational models, and enabling cross-study comparisons. Moreover, the increasing role of machine learning and data-driven modelling in neuroscience amplifies the need for open, well-curated, and high-quality datasets that bridge biological experiments and computational analysis.

**Fig. 1:**
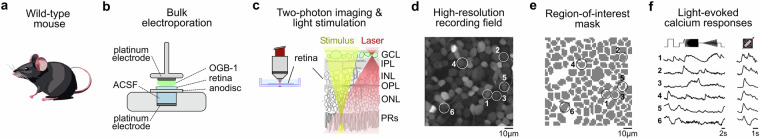
Data acquisition. (**a**) Illustration of a wild-type mouse with the genetic background *C57BL/6J*. (**b**) Schematic of the procedure to bulk electroporate a whole-mounted ex vivo retina. (**c**) Two-photon imaging of ganglion cell layer (GCL) somata in the whole-mounted ex vivo mouse retina. (**d**) Representative high-resolution recording field showing GCL somata loaded with Ca^2+^-indicator Oregon Green BAPTA-1 (OGB-1). (**e**) Semi-automatically generated region-of-interest (ROI) mask drawn on the 64 × 64 pixel scan field from the high-resolution field in (**d**) to extract individual cell responses. (**f**) Representative Ca^2+^-activity from cells in the GCL (white & black circles in (**d**) & (**e**)) in response to full-field “chirp” (left) and moving bar stimulus (right).

Here, we present the ALL-GCL dataset, a large-scale collection of functional recordings from over 80,000 cells in the ganglion cell layer (GCL) of the ex vivo mouse retina, obtained using two-photon Ca^2+^-imaging under standardised conditions (Fig. 1c). This dataset integrates recordings from multiple experimental studies spanning nine years, all performed with a shared battery of “fingerprinting” visual stimuli (Fig. 1f). Moreover, the dataset contains subsets of experiments with responses to a wide range of other stimuli, including natural movies. In the following, we describe the dataset in detail, summarise its composition and data quality, quantify batch effects, and demonstrate its potential for functional classification and comparative analyses.

The overarching goal of this work is to provide a rich and accessible resource for the vision science community. The ALL-GCL dataset enables large-scale analyses of retinal function, supports benchmarking of computational models, and may serve as a dataset for machine learning applications involving biological time-series data. We will continue to expand this resource with additional mouse lines (e.g., retinal degeneration mutants, such as *rd10*) and new stimulus protocols, creating a reference dataset for the functional organisation of the mouse GCL.

## Methods

### Input data

With the ALL-GCL dataset, we combine data recorded over a period of nine years. In total, the dataset contains 155 experimental sessions that were all originally collected for different studies:

- a study on colour vision by^11^,
- a machine-learning driven study on processing of natural scenes of RGCs by^12^,
- a study on the effects of nitric oxide on RGCs by^13^,
- a study on the functional degeneration of rd10 mice in which wild-type mice were used as a control group by^14^,
- unpublished work by Franke *et al*. (2018) on retinal processing of natural scenes,
- unpublished work by Cai *et al*. (2022–2024) on object-background segregation in the early visual system that was already presented at conferences^15^,
- and unpublished work by Kokat, Deja *et al*. (2022–2023) on suppressed-by-contrast RGCs in the early visual system that was already presented at conferences.

All data included share a standardised experimental procedure that features a set of “fingerprinting” stimuli, namely a full-field “chirp” and a moving bar, established by^7^. In addition, additional stimuli were presented depending on the scientific aim of the respective study (Fig. S1); these stimuli and responses are also included in the dataset. This large-scale aggregation enables systematic comparison of light-evoked activity across preparations and conditions, and represents a comprehensive resource for studying response diversity in the mouse GCL.

### Ethics statement

All animal experiments were conducted at the University of Tübingen and were performed according to the laws governing animal experimentation issued by the German Government as well as approved by the institutional animal welfare committee of the University of Tübingen (31.10.2016, CIN08/19M, CIN03/21M, CIN06/21M).

### Animals and preparation

For the pooled dataset, we used retinae (n = 227) from C57BL/6J mice (n = 155; JAX 000664, The Jackson Laboratory; Fig. 1a) of either sex between the age of postnatal day (P) P27 to P212. All animals were kept in the local animal facility and housed under the standard 12h/12h day/night cycle at 22^∘^C and a humidity of 55%.

The following procedures were carried out under very dim red (>650 nm) light. Before each imaging experiment, the animal was dark-adapted for >1h, then anesthetised with isoflurane (CP-Pharma) and sacrificed by cervical dislocation. Immediately after, the eyes were enucleated with a dorsal cut as orientation landmark and hemisected in carboxygenated (95% O_2_, 5% CO_2_) artificial cerebrospinal fluid (ACSF) solution containing (in mM): 125 NaCl, 2.5 KCl, 2 CaCl_2_, 1 MgCl_2_, 1.25 NaH_2_PO_4_, 26 NaHCO_3_, 20 glucose, and 0.5 L-glutamine at pH 7.4. Sulforhodamine-101 (SR101, 0.1 μM; Invitrogen) was added to the ACSF to reveal blood vessels and damaged ganglion cell layer (GCL) cells in the red fluorescence channel^16^. The carboxygenated ACSF was constantly perfused through the recording chamber at 4 ml/min and kept at  ~36 °C throughout the entire experiment.

After the dissection, retinae were bulk-electroporated with the synthetic fluorescent calcium indicator Oregon-Green 488 BAPTA-1 (OGB-1; hexapotassium salt; Life Technologies) (Fig. 1b)^17^. To electroporate the GCL, the dissected retina was flat-mounted with the GCL facing up onto an AnodiscTM (#13, 0.1 μm pore size, 13 mm diameter, Cytiva), and then placed between two 4 mm horizontal platinum disk electrodes (CUY700P4E/L, Nepagene/Xceltis). The lower electrode was covered with 15 μl of ACSF, while a 10 μl drop of 5 mM OGB-1 dissolved in ACSF was suspended from the upper electrode and lowered onto the retina. Then, nine electrical pulses ( ~9.2 V, 100 ms pulse width, at 1 Hz) from a pulse generator/wide-band amplifier combination (TGP110 and WA301, Thurlby Thandar/Farnell) were applied and then, the electroporated retina on the Anodisc was transferred into the recording chamber, whereby the dorsal edge of the retina pointed away from the experimenter. The retina was left there for  ~30 min to recover, as well as adapted to the light stimulation by displaying a binary dense noise stimulus (20 × 15 matrix, 40 × 40 μm^2^ pixels, balanced random sequence) at 5 Hz before the recordings started.

### Two-photon Ca^2+^-imaging

For the functional Ca^2+^-imaging experiments, a MOM-type two-photon microscope (Sutter Instruments/Science Products) was employed (Fig. 1c)^16,18^. The microscope was equipped with a mode-locked Ti:Sapphire laser (MaiTai-HP DeepSee, Newport Spectra-Physics) tuned to 927 nm (to optimally excite OGB-1), two photomultipliers serving as fluorescence detection channels for OGB-1 (HQ 510/84, AHF/Chroma) and SR101 (HQ 630/60, AHF), and a water immersion objective (CF175 LWD × 16/0.8W, DIC N2, Nikon, Germany). To acquire images, custom-made software (ScanM by M. Müller and T. Euler) running under IGOR Pro for Microsoft Windows (Wavemetrics) was used and time-lapsed 64 × 64 pixel image scans (100 × 100 μm) at 7.8125 Hz were taken. Routinely, the optic nerve position and the scan field position were recorded to reconstruct their retinal positions. High-resolution images (512 × 512 pixel images) were recorded to support region of interest (ROI) detection (Fig. 1d).

### Light stimulation

For the light stimulation of the retinal tissue, a digital light processing (DLP) projector (lightcrafter (LCr), DPM-E4500UVBGMKII, EKB Technologies Ltd) was used to display the visual stimuli through the objective onto the retina, whereby the stimulus was focused on the photoreceptor layer^19^. To enable differentially exciting the two spectral mouse cone types (PR1 and PR4, with peak sensitivities of 510 and 360 nm, respectively), the stimulator used two narrow bands at around  ~575 nm and  ~385 nm. This was implemented either by equipping the LCr itself with the respective LEDs and band-passing the projected image using a dual-band filter (390/576 Dualband, F59-003, AHF/Chroma) or by using a light-guide enabled LCr with an external, individually band-pass filtered green/UV LED unit (LEDs; green: 576 BP 10, F37-576; UV: 387 BP 11, F39-387; both AHF/Chroma; for details, see ref. ^19^).

In either configuration, the LEDs were synchronised with the scan retracing of the microscope. Stimulator intensity (as photoisomerisation rate, 10^3^ P^*^s^−1^ per cone) was calibrated to range from  ~0.5 (“black” image) to  ~20 for M-(LWS) and S-(SWS1) opsins, respectively. Steady illumination of  ~10^4^ P^*^s^−1^ per cone was present during the scan recordings due to the two-photon excitation of photopigments^16,18^.

Two types of light stimuli were used in all experimental sessions included in this dataset: A full-field “chirp” stimulus (with either 800 μm or 1,000 μm ∅; see details in ref. ^7^; Fig. S1a), and a bright bar (0.3 × 1 mm) moving along its long axis at 1 mm s^−1^ in eight directions to probe direction and orientation selectivity (Fig. S1c). The full-field “chirp” stimulus modulated light intensity across the whole stimulator intensity range (0%-100%), while the moving bar stimulus consisted of a bright bar at 100% intensity presented on a background at 0% intensity. These core stimuli were identical across all experiments, ensuring comparability of responses across recordings.

In addition, for subsets of experiments, we included complementary stimulus paradigms:

1. An interleaved “chirp” stimulus alternating between a small (100 μm ∅) and large (1,000 μm ∅) stimulation area across three trials (Fig. S1b);
2. A contrast saturation stimulus consisting of a central spot (100 μm ∅) presented together with a surrounding annulus (1,000 μm ∅) at 50% light intensity, with the centre intensity modulated by sinusoidal luminance fluctuations at varying contrast levels (10%, 20%, 40%, 60%, 80%, and 100%) across three trials (Fig. S1d),
3. A spot size stimulus consisting of circular spots with diameters ranging from 20 μm to 500 μm, each presented for 1 s, interleaved by blank periods of 2 s, and repeated across three trials (Fig. S1e);
4. A binary dense noise stimulus (20 × 15 matrix, (40 μm)^2^ pixels, balanced random sequence; 5 Hz) (Fig. S1f);
5. A spatially shifted (“shifty”) noise stimulus using the same binary checkerboard design (40 μm pixel size) but additionally jittering the spatial position of the entire pattern randomly between frames on a 10 μm grid, following the approach described by^20^ (Fig. S1g). Note that due to an implementation detail, the spatial shift was only applied during the last three quarters of each frame (when the trigger signal was low), while the pattern remained stationary during the first quarter of each frame (when the trigger signal was high);
6. A blue and green centre-surround flicker stimuli consisting of a central spot (∅ 250 μm) and a surrounding annulus (inner ∅ 250 μm, outer ∅ 700 μm) driven by temporally varying intensity sequences at 5 Hz (Fig. S1h);
7. Natural movies generated from footage recorded by^21^ and first used in^12^ (Fig. S1i).

Stimulus timing was consistently aligned across recordings using time stamps (“stimulus triggers”) embedded into the imaging files. These triggers mark key events, such as the beginning of a trial (see red vertical lines in Fig. S1). Each stimulus was associated with a fixed number of triggers (e.g., two for the chirp stimulus and eight for the moving bar stimulus). All trigger information was exported and is available in the data repository, enabling precise alignment of recorded responses with the underlying stimulus structure. Light stimulus centre and scan field centre were aligned for all stimuli. Before a stimulus was presented, the baseline was recorded after the laser started scanning for at least 10 s to allow any immediate laser-induced effect on the retinal activity to have subsided^11,16,18^.

### Data analysis

#### Software

Image extraction was performed using IGOR Pro. All analyses were performed in Python and the results were stored and organised in a custom DataJoint database^22^.

#### Regions of interest

Regions of interest (ROIs), which correspond to the somata of individual cells in the GCL, were manually drawn in the input data using IGOR Pro (Fig. 1e). Some of the input data originally excluded cells with strong SR101 filling from the ROI masks because it was assumed that SR101 filling correlated with cell death. Here, however, we wanted to include all cells in the ROI masks. To this end, we added previously unlabelled cells to the ROI masks as follows: For each field, we generated a new temporary ROI mask using Cellpose 3^23^. If any new ROIs were missing from the manually drawn ROI masks (defined as having a maximum intersection-over-union of 0.1 with any other ROI), we added them to the manual ROI mask after clipping any overlapping or directly adjacent pixels.

#### Preprocessing

Ca^2+^-traces were extracted from individual ROIs as the mean over all pixels in the ROI for each imaging frame. These raw traces were detrended by subtracting a smoothed version *r*_*smooth*_ of the trace from the raw one. Detrending was necessary to remove slow drifts in the signal that were unrelated to the light-induced response. The smoothed trace *r*_*smooth*_ was computed by applying a Savitzky-Golay filter^24^ of 3^*rd*^ polynomial order and a window length of 60 s (for “chirp”, moving bar) and 120 s (for the other stimuli) using the Python SciPy implementation^25^.

$${r}_{detrend}={r}_{raw}-{r}_{smooth}$$

For the “chirp” and moving bar stimulus, we further computed response averages from the detrended traces as means over repetitions. In the case of the moving bar stimulus, the response average was reduced to the response to the preferred motion direction of the cell (for details, see ref. ^7^). Finally, response averages were normalised by first subtracting the baseline activity (computed as the mean over the first second preceding stimulus onset), and then by dividing by the maximum amplitude ${\max }_{t}(| r(t)| )=1$. This normalisation was performed independently for each ROI, stimulus, and condition. Direction and orientation selectivity – qualified as DSi and OSi, respectively – were computed from the moving bar responses using a permutation test as described in^7^. Also the On-Off index (OOi), which describes the response polarity preference of a cell to light increments and/or decrements was computed as described before^7^. For the natural movie responses, we additionally used the spike-inference algorithm CASCADE to estimate spike rates from the preprocessed traces^26^.

#### Response quality

To quantify the response quality of individual ROIs, we computed a quality index (QI) for repeated stimuli such as the moving bar (QI_MB_) and full-field “chirp” (QI_chirp_):

$$QI=\frac{Var\,{[{\langle C\rangle }_{r}]}_{t}}{{\langle Var{[C]}_{t}\rangle }_{r}}$$

where *C* is the *T* by *R* response matrix (time samples by stimulus repetitions) and ${\langle \rangle }_{x}$ and *V* *ar*[]_*x*_ denote the mean and variance across the indicated dimension *x*, respectively. For the moving bar, the QI was computed for all directions independently using the direction with the largest QI as QI_MB_. Cells with either QI_*MB*_ > 0.6 or QI_chirp_ > 0.35 were considered *responsive* in the following analysis. Quality index thresholds were chosen based on values used in related work (e.g.,^7,13,14^) and adapted in this dataset to ensure a balance between data quality and comparability. A recording field is considered responsive if at least a third of the ROIs have a QI_MB_ > 0.6. Note that the dataset contains all recorded cells, including those not meeting these quality criteria, allowing users to apply alternative thresholds depending on their specific analysis requirements.

#### Classification of functional ganglion cell layer groups

For the functional classification of GCL types, we built on a previously published GCL classifier^13,27^, which is a *scikit* − *learn* random forest classifier trained on previously published GCL responses and functional cell group labels^7^. As in the previous version, the classifier predicts a functional group label for each cell independently using the following inputs:

- 20 features from the normalised response average to the full-field “chirp”,
- 8 features from the normalised response average to the moving bar projected to the preferred direction,
- a p-value from a permutation test for direction selectivity,
- and the ROI sizes as a proxy for soma-size.

The features of the “chirp” and moving bar responses were extracted using the sparse PCA embedding matrix that was used in^7^ for clustering. We split the labelled data (n = 7,979 ROIs) of^7^ into a train set (90%) and held-out test set (10%) using stratification by cluster label, ensuring that the relative frequency of each cell group label was approximately preserved in both the training and test set. We used the original 75 cluster labels from that previous study as the classifier output. These labels are derived from the established functional classification framework and were not intended as ground truth, but rather as a widely used reference taxonomy. Accordingly, the reported accuracy reflects the agreement of the classifier with this reference classification on held-out data, rather than an independent validation against an external ground truth. Hyperparameters of the random forest classifier, such as the number of trees, maximum tree depth, number of features sampled per split, were tuned using a cross-validated grid search. Using the final set of hyperparameters, the random forest classifier underwent uncertainty calibration (using 5-fold cross-validated probability calibration with logistic regression) and was then evaluated on the held-out test-set achieving an accuracy of 0.764 (at a chance level of 0.013). Finally, using the same hyperparameters, the classifier was re-trained on the full^7^ dataset, i.e. including the test data.

As this model is used solely for prediction rather than validation, merging the test set into the training data ensures the classifier learns from the full distribution of the original study. The classifier outputs a probability distribution over the 75 cluster labels. In a post-processing step, we merged the 75 clusters to the 46 functional groups G_1_ to G_46_ as in^7^, summing the respective classifier output probabilities. A cell was assigned to the group for which the classifier predicted the largest probability; we defined this largest probability as the classification confidence of the uncertainty-calibrated classifier. To predict group labels for new data, we replicated the preprocessing of the original study as closely as possible and applied it to the new data.

Note that within the present dataset, the “chirp” stimulus was identical in its temporal structure. The temporal structure of the chirp stimulus is provided in the data repository, allowing direct alignment of responses to stimulus timing. However, the “chirp” used in^7^ was slightly different in its timing properties compared to the “chirp” used in this dataset. To enable consistent cross-dataset comparison, we warped the time of the new data to maximise agreement with the original dataset. This temporal warping was applied solely for cross-dataset alignment and does not affect the raw or processed data provided in the present dataset. The code for the GCL classifier can be found in the corresponding GitHub repository (github.com/eulerlab/gcl_classifier).

#### Receptive field estimation

We mapped receptive fields (RFs) of cells using the RF Python toolbox *RFEst*^28^, following the procedure in^7^ with few modifications. We computed RFs for the responses to the dense noise stimulus, the shifty noise stimulus, and the blue and green centre–surround flicker stimuli. We computed the temporal gradients of the Ca^2+^-signals as a proxy for calcium influx, from the detrended traces and clipped negative values:

$$\mathop{c}\limits^{.}=\max \left(0,{\mathop{r}\limits^{.}}_{detrend}\right)$$

We clipped negative gradients because only positive deflections reflect calcium influx driven by spiking activity, whereas negative gradients primarily reflect the slower dissociation of calcium from the indicator and would contribute spurious anti-correlations to the RF estimate. The stimulus ***S***(*x*, *y*, *t*), originally presented at 5 Hz, and the clipped temporal gradients $\mathop{c}\limits^{.}$, originally recorded at 7.8125 Hz, were upsampled to 20 Hz. For the gradients we used linear interpolation; for the stimulus we held each frame constant until the next frame was displayed, since this reflects what was actually shown on screen. From this we computed the gradient-triggered average stimulus:

$${\boldsymbol{F}}(x,y,\tau )=\frac{1}{{\sum }_{i=1}^{M}\,\mathop{c}\limits^{.}({t}_{i})}{\sum }_{i=1}^{M}\,\mathop{c}\limits^{.}({t}_{i}){\boldsymbol{S}}(x,y,{t}_{i}+\tau )$$

where ***S***(*x*, *y*, *t*) is the stimulus, *τ* is the lag ranging from approximately  − 1.35 to  + 0.45 seconds, and *M* is the total number of (upsampled) time samples.

For dense and shift noise, we smoothed these raw RFs using a 3  × 3 pixel and 1 pixel standard deviation Gaussian window for each lag. Then we decomposed the RF into a temporal (*F*_*t*_(*τ*)) and spatial (***F***_*s*_(*x*, *y*)) component using singular value decomposition and scaled them such that $\max (| {F}_{t}| )=1$ and $\max (| {{\boldsymbol{F}}}_{s}| )=\max (| {\boldsymbol{F}}| )$.

We assessed RF quality with two complementary metrics. First, we computed the space-time separability index:

$$Q{I}_{{\rm{RF}}}=1-\frac{Var\,[{\boldsymbol{F}}(x,y,\tau )-{F}_{t}(\tau ){{\boldsymbol{F}}}_{s}(x,y)]}{Var\,[{\boldsymbol{F}}(x,y,\tau )]}$$

which quantifies how well the RF is captured by a single space-time-separable component, and is therefore a prerequisite for the spatial RFs ***F***_*s*_(*x*, *y*) shown in the figures to be meaningful. Second, to provide a quality measure that does not assume space-time separability, we computed a size-corrected signal-to-noise ratio. We estimated the noise standard deviation *σ*_noise_ as the pooled standard deviation of ***F***(*x*, *y*, *τ*) across all spatial locations within an baseline window of 0.2 s at the most positive lags (*τ* close to  + 0.45 s), where no stimulus-driven signal is expected. The raw peak *z*-score was defined as ${z}_{{\rm{peak}}}=\mathop{\max }\limits_{x,y,\tau }\left|{\boldsymbol{F}}(x,y,\tau )\right|/{\sigma }_{{\rm{noise}}}$. Because the expected maximum of $\left|z\right|$ over *T* lags and *S* spatial locations of approximately independent Gaussian noise scales as $\sqrt{2\,\log (TS)}$, we report the size-corrected SNR

$${\rm{SNR}}=\frac{{z}_{{\rm{peak}}}}{\sqrt{2\,\log (TS)}},$$

where *T* is the number of time lags and *S* is the number of spatial pixels. This statistic has a null distribution centred near 1 regardless of *T* and *S*, and is therefore not inflated by padding the stimulus with noise-only pixels outside the RF. Only RFs with *QI*_RF_ > 0.4 were used for the analyses requiring a spatial RF; SNR values are provided alongside the RFs in the dataset for users who wish to apply alternative quality criteria.

For each spatial RF ***F***_*s*_, we fit a 2D Gaussian using the Python package *astropy*^29^. The area covered by two standard deviations of this Gaussian fit was used as the RF size.

#### Estimating batch effects

To separate biological signals from potential effects induced by confounding variables in the dataset – so-called batch effects – we trained classifiers to predict metadata attributes, such as the animal’s sex or the experimenter, solely from the recorded neural activity.

We first filtered the dataset by selecting traces with the following response criteria: QI_chirp_ > 0.35 or QI_MB_ > 0.6. Based on a random seed, the filtered dataset was split into training (80% of cells) and test (20% of cells) sets based on recording dates. This ensured that the test set evaluated performance on previously unseen dates, preventing data leakage. To avoid class imbalance, we downsampled the majority class to ensure a random classifier would achieve an average accuracy of 50% assuming two classes.

To create the input features of the classifier, we concatenated the preprocessed “chirp” (259 time points) and moving bar responses to the preferred direction (32 time points) into a 291-dimensional feature vector. Then we began by training a model to distinguish between the three recording setups. Because distinct setups proved identifiable, we restricted all subsequent experiments to setup ID 1, the setup used for most recordings. For all remaining predictions, we binarised the metadata attributes for a binary classification task. Data points falling between binary thresholds were excluded to ensure separation. We trained classifiers to predict the sex of the animal (female/male), scan field ID (first/third field), retinal location (ventral/dorsal relative to the optic nerve, excluding cells within 100 μm of the optic nerve), and age (≤10 weeks vs. >15 weeks, that is, younger than P71 vs. older than P104). To assess experimenter effects, we trained models to differentiate between pairs of experimenters.

To further estimate recording-session-specific effects independent of experimenter-related variability, we performed a binary date-classification task on recordings from the same experimenter. Since each recording date corresponds to a separate session, above-chance prediction of the date from neural activity indicates session-specific variability, although residual biological differences cannot be fully ruled out. The analysis was restricted to the first experiment (i.e., the first retina of a session) and its first two recorded recording fields. Date pairs were included only if both dates contained at least 30 quality-filtered cells per field, animal age was at least 4 weeks (≥P28) and differed by at most 10 weeks, and recordings were performed by the same experimenter on setup 1. For each date pair, a classifier was trained on cells from one scan field and evaluated on the other. Class balance was enforced independently in the training and test sets via downsampling, yielding a chance level of 50%.

For all tasks, we used a high-capacity random forest classifier (1,000 estimators, maximum depth of 50). To ensure robustness, we performed 20 independent training runs with different random seeds for the train/test split.

#### Topographic mapping

To compute cell-type specific spatial density maps, we restricted the dataset to responsive cells with information about their location. Next, we computed a 2D Gaussian kernel-density estimate (KDE) for each cell-type *ct* independently *d*_ct_(*x*, *y*). The KDE were evaluated on a regular grid of 51 × 51 points spanning 2 mm from the optic disc centre. We normalised the cell-type specific estimates using a KDE map over all cell types:

$${d}_{{\rm{ct-norm}}}({x}_{i},{y}_{j})={d}_{{\rm{ct}}}({x}_{i},{y}_{j})/{d}_{{\rm{all}}}({x}_{i},{y}_{j})$$

for all *x*_*i*_ and *y*_*j*_ on the grid.

## Data Record

The dataset (v2.0) generated in this study is publicly archived via Zenodo, which provides a persistent DOI for citation and versioning. The underlying dataset files are hosted on the Hugging Face Hub and can be accessed at huggingface.co/datasets/eulerlab/all-gcl^30^. The Zenodo record serves as the canonical citation entry and links to the corresponding Hugging Face repository. The data are standardised according to the Neurodata Without Borders (NWB;^31^) specifications to ensure inter-operability and long-term accessibility.

The dataset is organised hierarchically by experimental session. The root directory contains folders for each session (naming convention: Session_Experimenter_ID_Date), which branch into subfolders for specific experiments and anatomical locations (e.g., experiment_1_LR for **L**eft **R**etina). Inside these subdirectories, individual .nwb files represent distinct recording fields (e.g., field_GCL1.nwb), each corresponding to an individual imaging scan field within a session. Additionally, a single aggregated summary table (all_GCL_table.parquet) is provided at the root level, containing extracted features for all cells across all experiments to facilitate rapid analysis without parsing individual .nwb files.

Each .nwb file includes both the raw Ca^2+^-imaging data and the processed results. The internal structure follows the NWB standard, with raw acquisition data stored under acquisition/ (organised by stimulus condition: Full-field “chirp”, moving bar, dense noise, etc.) and processed results under processing/. Key metadata and analysis outputs include:

- *General metadata*. Experimental details including animal age, sex, genetic line, eye orientation, and anatomical position (ventral-dorsal/temporal-nasal coordinates relative to the optic disc).
- *Functional classification*. Functional cell group labels (cluster IDs 1-75, group IDs 1-46, supergroup IDs 1-6), confidence scores, and probability distributions aligned with the^7^ classification scheme.
- *Stimulus responses*. Preprocessed response traces and quality indices (Signal-to-Noise Ratio) for all stimuli (see Fig. S1). Trial-wise responses and corresponding averaged traces are both included.
- *Feature indices*. Computed functional metrics, including DSi, OSi, and OOi (see above).
- *Receptive fields*. Spatio-temporal RFs derived from dense noise and shifty noise stimuli, including spatial parameters (RF diameter, Gaussian fit quality) and temporal dynamics (lag, transience). The temporal dynamics together with the RFs were also included for the centre-surround colour flicker stimuli.

Detailed descriptions of all variable names, column headers, and unit specifications are provided in the README.md file accompanying the data repository.

## Data Overview

**Fig. 2:**
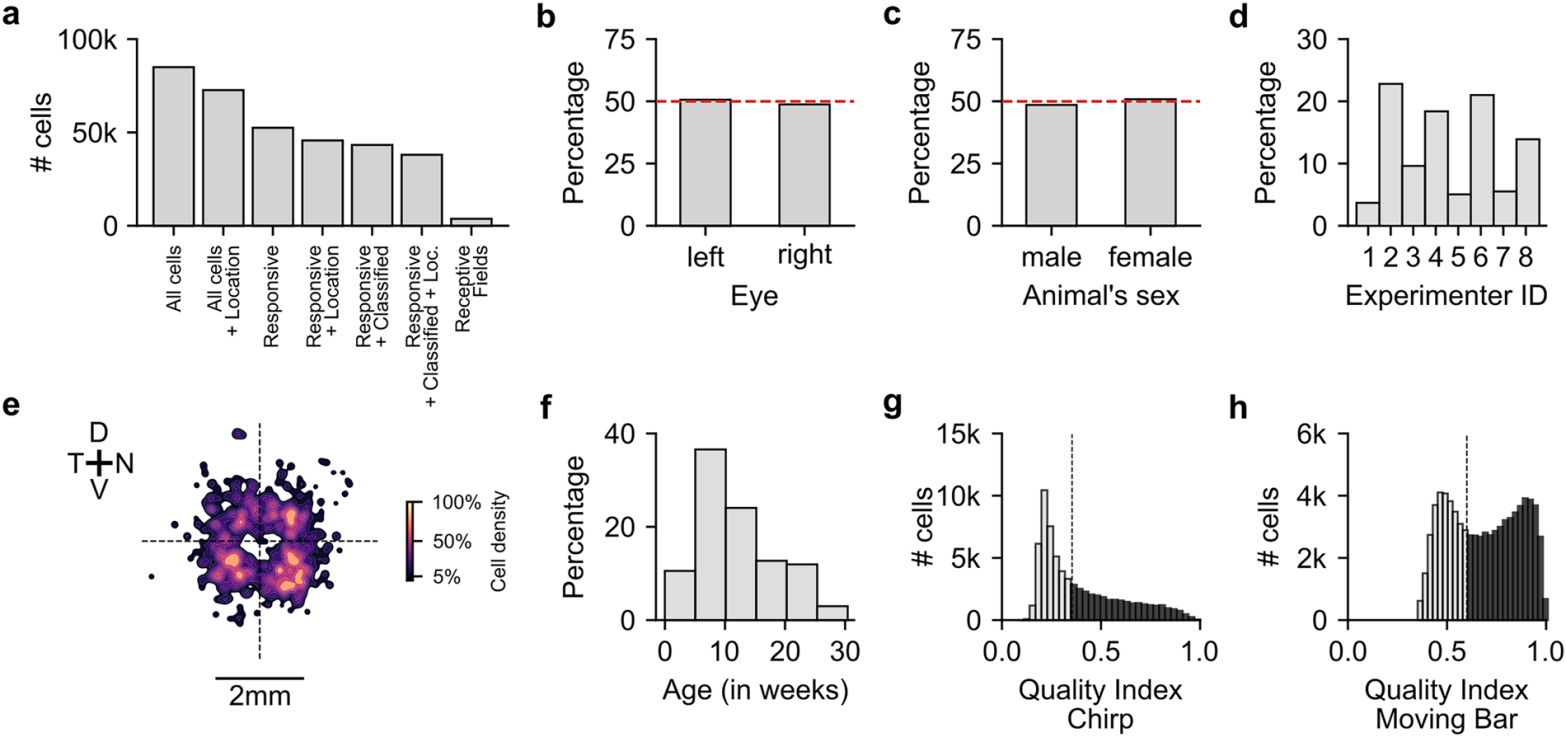
Data overview. (**a**) Number of recorded cells across different selection and response criteria. (**b,c**) Distribution of recordings across eyes (left vs. right) and animal sex (male vs. female), respectively. (**d**) Proportion of cells recorded by each experimenter. (**e**) Spatial distribution of recorded cells within the retina, shown as a density map (D, dorsal; V, ventral; T, temporal; N, nasal); Scale bar: 2 mm. (**f**) Age distribution of animals used for all recordings. (**g,h**) Distribution of response quality indices for full-field “chirp” (**g**) and moving bar (**h**) stimuli, with dashed lines indicating quality thresholds.

In total, the ALL-GCL dataset contains responses from more than 80,000 GCL somata, with  ~85% having information about their exact retinal recording location (Fig. 2a,e). The spatial distribution of recordings covers almost the entire retinal surface, with a slight sampling bias toward ventral regions (Fig. 2e). To ensure data quality, responses were evaluated using a quantitative quality index (QI), with  ~65% exceeding the selected response criterion (QI_chirp_ ≥ 0.35 or QI_MB_ ≥ 0.6; Fig. 2g,h; see Methods for details).

The dataset is well balanced with respect to eye location and sex: approximately half of the cells were recorded from left eyes and the other half from right eyes (Fig. 2b), and recordings are similarly split between male and female animals (Fig. 2c). Recordings were contributed by eight experimenters (Fig. 2d). The animals used for recordings spanned a broad age range from P27 to P212 (Fig. 2f), thus encompassing both juvenile and mature animals.

Together, these data provide a large-scale, well-balanced, and high-quality foundation for type-specific analyses of light-evoked GCL activity. The scale and consistency of the ALL-GCL dataset enable reliable cross-comparisons across experiments, forming the basis for subsequent analyses.

### Functional GCL group classification

To understand how the retina segregates visual information into parallel output channels, one can identify cell groups within the GCL sharing functional response features. Using this approach,^7^ showed that the GCL comprises approximately 46 functional groups, including 32 retinal ganglion cell (RGC) groups and 14 displaced amacrine cell (dAC) groups. Building on this classification framework, we sought to extend group-specific classification to the ALL-GCL dataset to enable systematic, large-scale analyses of functional diversity across recordings.

For this purpose, we employed an updated version of the previously published GCL-group classifier (^27^; for details, see Methods). In brief, the supervised classifier was trained on features extracted from full-field “chirp” and moving bar responses, complemented by soma size and direction-selectivity parameters from^7^. The group labels from that study served as reference group labels. We emphasise that this procedure maps cells in the ALL-GCL dataset onto an existing reference taxonomy, rather than defining a new classification scheme. In particular, the commonly used 46 functional groups follow^7^, where finer response clusters are consolidated into reproducible functional classes. To predict cell groups in the ALL-GCL dataset, each recorded response was projected into the same feature space, and the classifier assigned a predicted group label along with an associated confidence score. Depending on the desired analysis level, each cell is annotated with three hierarchical labels: (1) functional group, (2) functional supergroup (i.e., ‘Off’, ‘On-Off’, ‘Fast On’, ‘Slow On’, ‘Uncertain RGC’, ‘dAC’), and (3) GCL class (RGC, dAC), each accompanied by its corresponding confidence score.

**Fig. 3:**
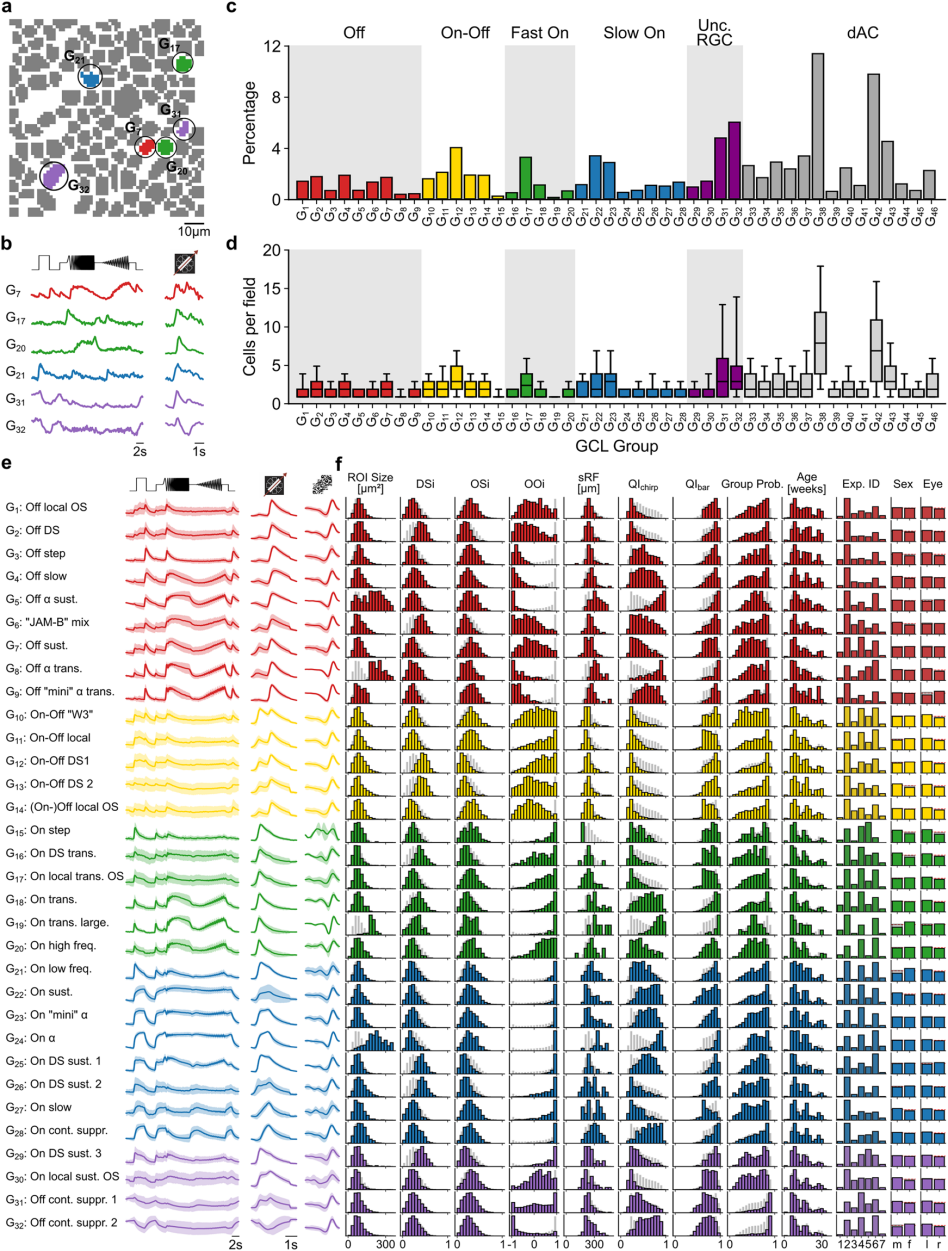
Functional classification of GCL somata. (**a**) Semi-automatic drawn region-of-interest (ROI) mask on top of the recording field from Fig. 1d with examples of functionally classified ganglion cell layer (GCL) groups. (**b**) Representative average “chirp” and moving bar responses from the recording field shown in panel a, colour-coded according to their classified functional group. (**c**) Distribution of cell groups across functional supergroups, separated into ‘Off’, ‘On-Off’, ‘Fast On’, ‘Slow On’, ‘Uncertain RGCs’, and displaced amacrine cells (dACs). (**d**) Average number of cells per recording field for each functional group. (**e**) Mean “chirp”, moving bar responses, and temporal receptive field for all identified GCL groups, organised by functional group and colour-coded consistently with panels b-d. (**f**) Corresponding distribution of key physiological and experimental parameters for each group, including ROI size, direction selectivity index (DSi), orientation selectivity index (OSi), On-Off index (OOi), spatial receptive field area (sRF), “chirp” and moving bar response quality indices, and metadata (age, experimenter ID, sex, eye).

Applying this refined classifier to the ALL-GCL dataset yielded group predictions for all recorded cells, spanning the full range of known functional groups (Fig. 3a–c). The resulting population structure closely reflects the distribution reported by^7^, with comparable proportions of ‘Off’, ‘On-Off’, ‘Fast On’, ‘Slow On’, ‘Uncertain RGCs’, as well as ‘dACs’ (Fig. 3c). For each group, we also computed the average cell count per field with certain cells being more frequent than others (Fig. 3d). Representative average responses to the “chirp” and moving bar stimuli for selected groups illustrate the diversity of temporal and directional tuning among identified GCL types (Fig. 3b,e, Fig S2a).

To further characterise each group, we computed and added the distributions of key physiological and morphological parameters, including ROI size (reflecting soma size in μm^2^), DSi, OSi, OOi, spatial RF size (μm), quality indices for “chirp” and moving bar, probability of the group label classification, age range, experimenter ID, animal’s sex, and eye (Fig. 3f, Fig S2b). Across groups, these features followed characteristic distributions that matched previously described response motifs. The consistent mapping of these properties validates the reliability of the classifier across the full dataset.

Importantly, the predicted group, supergroup, and class labels, as well as the corresponding confidence scores are made available as part of the ALL-GCL dataset. These labels provide users with an immediately usable framework for querying, filtering, and analysing GCL responses by functional group. Together, they enrich the dataset with biologically meaningful structure and enable researchers to perform group-specific analyses without additional preprocessing.

## Technical Validation

### Validating RGC groups through their topographical distributions

Previous studies have characterised topographic distributions of RGC types using spatial transcriptomics (e.g.,^9,32,33^), immunohistochemistry/transgenic lines (e.g.,^34–36^), and functional recordings for a subset of types (e.g.,^7,37,38^). For several groups, particularly alpha RGCs^39^, functional identities align well with molecular and anatomical descriptions^8^. These prior findings provide a reference for validating the spatial organisation present in the ALL-GCL dataset.

**Fig. 4:**
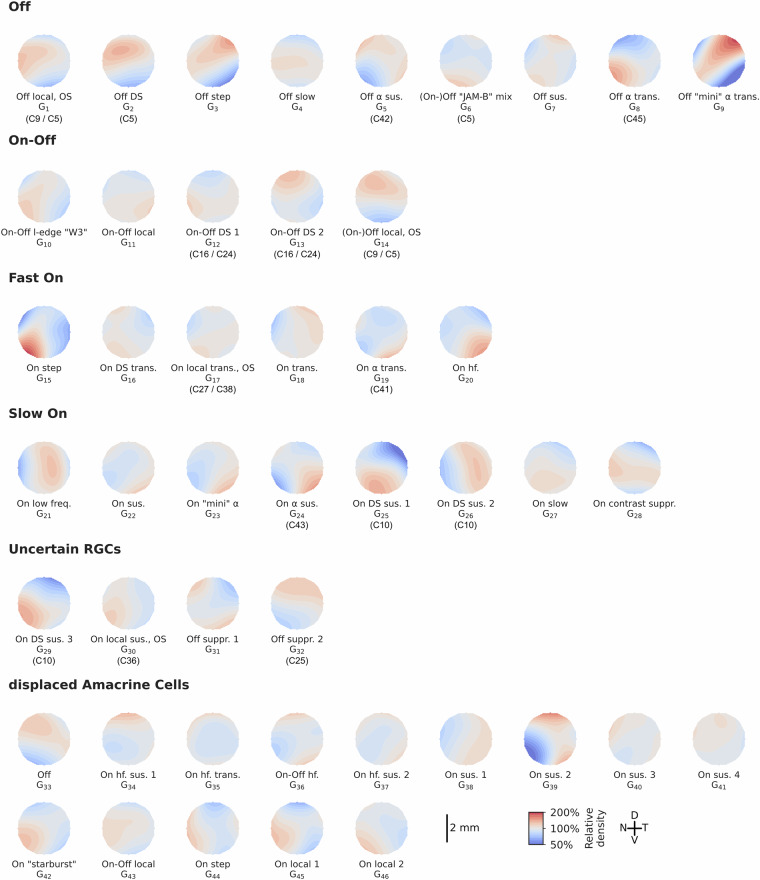
Topographic distribution of functional GCL groups. Spatial distribution maps of all identified functional GCL groups across the retina. Each circle represents the relative density of a specific functional group, with colour indicating the normalised cell density (red: higher density, blue: lower density). Functional groups are arranged by response class: ‘Off’, ‘On-Off’, ‘Fast On’, ‘Slow On’, ‘Uncertain RGCs’, and ‘displaced amacrine cells’. D, dorsal; V, ventral; T, temporal; N, nasal. Scale bar, 2 mm. Where clear correspondences exist, functional groups are additionally annotated with transcriptomic cluster identities (C1-C45) from^9^, based on high- and medium-confidence mappings provided by^8^.

To assess consistency with earlier studies, we computed spatial density maps for all 46 functional GCL groups (Fig. 4), expressed as relative density normalised with respect to the density of all cells. At the level of functional supergroups, the observed distributions agreed with established large-scale patterns: ‘Off’ groups (G_1_-G_9_) showed dorsal enrichment, while ‘Fast On’ and ‘Slow On’ groups (G_15_-G_19_ and G_20_-G_28_) were enriched ventrally. ‘On-Off’ groups (G_10_-G_14_), as well as ‘Uncertain RGCs’ (G_29_-G_32_) and ‘dACs’ (G_33_-G_46_), exhibited weaker or more heterogeneous gradients, consistent with earlier anatomical and functional observations.

For individual groups with well-documented topography, the maps reproduced expected trends. The On alpha sustained group (G_24_) showed temporal-ventral enrichment in agreement with previous studies^32– 35,40^, and the On contrast suppressed group (G_28_) showed a naso-temporal bias consistent with recent functional findings^12^. The overall complementarity of dorsal ‘Off’ and ventral ‘On’ distributions matches established retinal specialisations^11,41^.

To facilitate comparison with spatial transcriptomic studies, we additionally annotate selected functional groups with corresponding transcriptomic cluster identities (^9^; C1-C45) where agreement across modalities is well established. These mappings are not one-to-one for all cell types and are therefore restricted to high- and medium-confidence correspondences based on^8^.

Together, these correspondences demonstrate that the spatial organisation of functional groups in the ALL-GCL dataset aligns well with published molecular, anatomical, and functional topographies, providing technical validation for the group assignments.

### Quantifying batch effects in the dataset

Variability introduced by extraneous, non-biological factors — commonly referred to as batch effects — can obscure or confound meaningful biological signals in datasets. Such effects may arise from subtle differences in experimental conditions, handling procedures, or systematic variation across data collection sessions. For example, Zhao *et al*.^42^ reported subtle batch effects in retinal bipolar cell recordings, including changes in response kinetics likely induced by temperature fluctuations. Similar heterogeneity across retinas has also been reported using spike-based analyses^43^.

To assess the extent of such influences in the ALL-GCL dataset, we examined six key metadata parameters: recording setup ID, experimenter identity, scan field ID (first or third recording field), retinal location (ventral or dorsal), animal sex (male or female), and age (juvenile or adult). Among these, sex, age, and retinal location represent biological covariates, whereas setup ID and experimenter identity capture technical variability. The scan field parameter reflects a combination of both biological and technical factors.

**Fig. 5:**
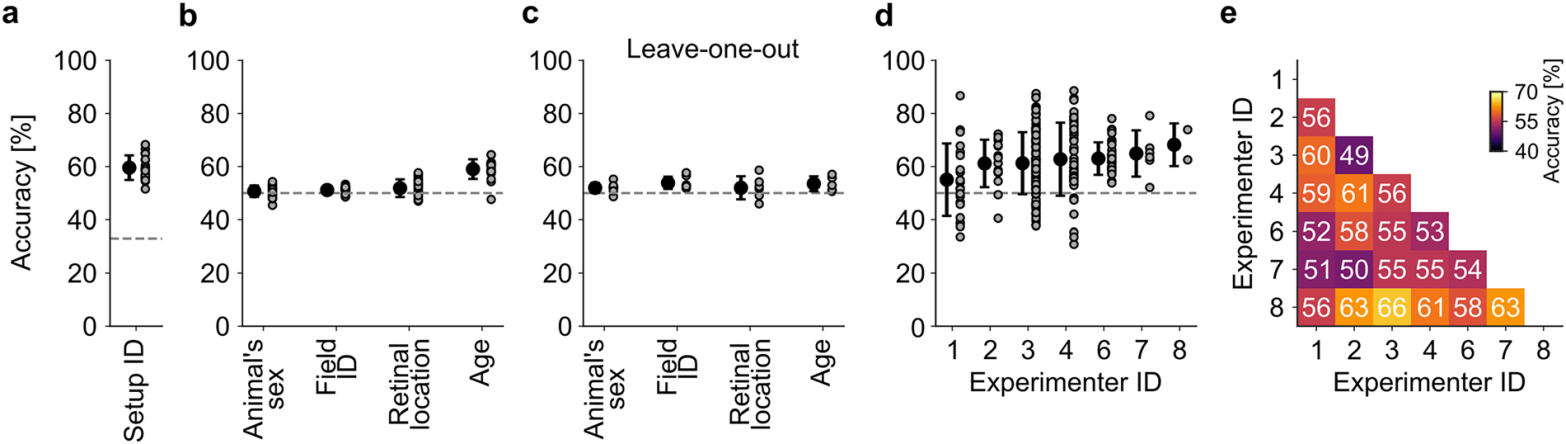
Predictability of experimental and biological variables from GCL cell features. (**a**) Prediction accuracy for the experimental setup ID using a random forest classifier. Each point represents an independent random seed and dataset split. Dashed line indicates chance level (33.3%). (**b**) Prediction accuracy for various metadata categories using a random forest classifier. Each point represents an independent random seed and dataset split. Dashed line indicates chance level (50%). (**c**) Prediction accuracy using a leave-one-experimenter-out training scheme, where models were trained on all but one experimenter and tested on the held-out one. Error bars represent mean  ± SD across dataset splits. (**d**) Prediction accuracy for recording session identity across experimenters, assessing session-related batch effects while controlling for other variables. Each point represents an independent random seed and dataset split. Dashed line indicates chance level (50%). (**e**) Cross-experimenter prediction accuracy matrix, showing pairwise classification accuracy when discriminating between experimenters. Colour scale indicates accuracy (40–70%).

To quantify how strongly these variables are reflected in functional responses, we trained random forest classifiers to predict each metadata parameter from response features (Fig. 5). Prediction of recording setup ID yielded accuracies above chance (59.6%  ± 4.6%; Fig. 5a), indicating detectable setup-related differences. Consequently, for subsequent analyses, we restricted the dataset to the setup with the largest number of recordings to minimise confounding effects from setup variability.

Prediction accuracies for animal sex, scan field ID, and retinal location were close to chance level (Fig. 5b), suggesting that these factors introduce little systematic structure into the functional responses. Age could be predicted slightly above chance when pooling all data (59.0%  ± 3.7%), but this effect weakened under a leave-one-experimenter-out validation scheme (Fig. 5c), indicating that the apparent age-related signal is largely driven by experimenter-associated structure rather than reflecting robust biological differences.

We next assessed recording-session-associated variability by testing whether recording date could be predicted from responses within each experimenter (Fig. 5d). For each experimenter, classifiers were trained to discriminate between recording sessions based on functional response features. This analysis revealed above-chance prediction accuracies (typically  ~55–70%), indicating measurable session-related structure in the data. Because recording date can be partially confounded with biological variables such as animal identity, retinal location, and sampling differences across experiments, we interpret this result as evidence for session-associated dataset structure rather than a purely technical batch effect.

To evaluate experimenter-specific variation, we trained classifiers to predict experimenter identity from functional responses while restricting the analysis to recordings obtained with the same setup (Fig. 5e). Some pairs of experimenters could be distinguished (up to  ~66% accuracy), whereas others remained close to chance level. These results indicate subtle but detectable experimenter-associated structure in the dataset.

Taken together, accuracies near chance for sex, retinal location, scan field ID, and age (under cross-experimenter validation) indicate that these parameters introduce little systematic structure into the functional responses. In contrast, above-chance accuracies for setup, certain experimenters (~60–66%), and recording session reveal measurable sources of variability in the dataset. As these factors may be partially confounded with biological covariates across experiments, we interpret them as indicators of dataset structure rather than isolated technical batch effects. Overall, these analyses suggest that the ALL-GCL dataset is broadly consistent across recordings, while also providing quantitative estimates of metadata-associated variability that should be considered in downstream analyses.

## Supplementary information

Supplementary Information

## Data Availability

The dataset is publicly available via Zenodo at 10.5281/zenodo.21789286, which provides a persistent identifier for citation and versioning. Due to the dataset size, the full dataset is hosted on Hugging Face and can be accessed at https://huggingface.co/datasets/eulerlab/all-gcl huggingface.co/datasets/eulerlab/all-gcl^30^. The dataset was exported in two formats: (1) a compact *p**a**n**d**a**s*. *D**a**t**a**F**r**a**m**e* in .parquet format, and (2) detailed single-session exports in the Neurodata Without Borders (NWB) format^31^. All data are publicly accessible without restriction.

## Code availability

The DataJoint database was derived from the Python package *d**j**i**m**a**g**i**n**g*. The version used for this dataset can be found at https://github.com/eulerlab/djimaging/releases/tag/all-gcl-v0.1.0 github.com/eulerlab/djimaging/releases/tag/all-gcl-v0.1.0. It includes all the code that was used to preprocess the data. The exact schema definition, i.e. which *d**j**i**m**a**g**i**n**g* tables have been used and how, and all code for figure generation is available from https://github.com/eulerlab/all-GCL-manuscript. The GCL classifier is available from https://github.com/eulerlab/gcl_classifier/.
