# Supplementary material for: A large-scale dataset of functional mouse ganglion cell layer responses
Source: Sci Data. 2026 Aug 11;13:1170. doi: 10.1038/s41597-026-08026-0 (PMC13462126; doi:10.1038/s41597-026-08026-0)
Supplement: Supplementary file 1 [file 41597_2026_8026_MOESM1_ESM.pdf]

Supplementary Figures

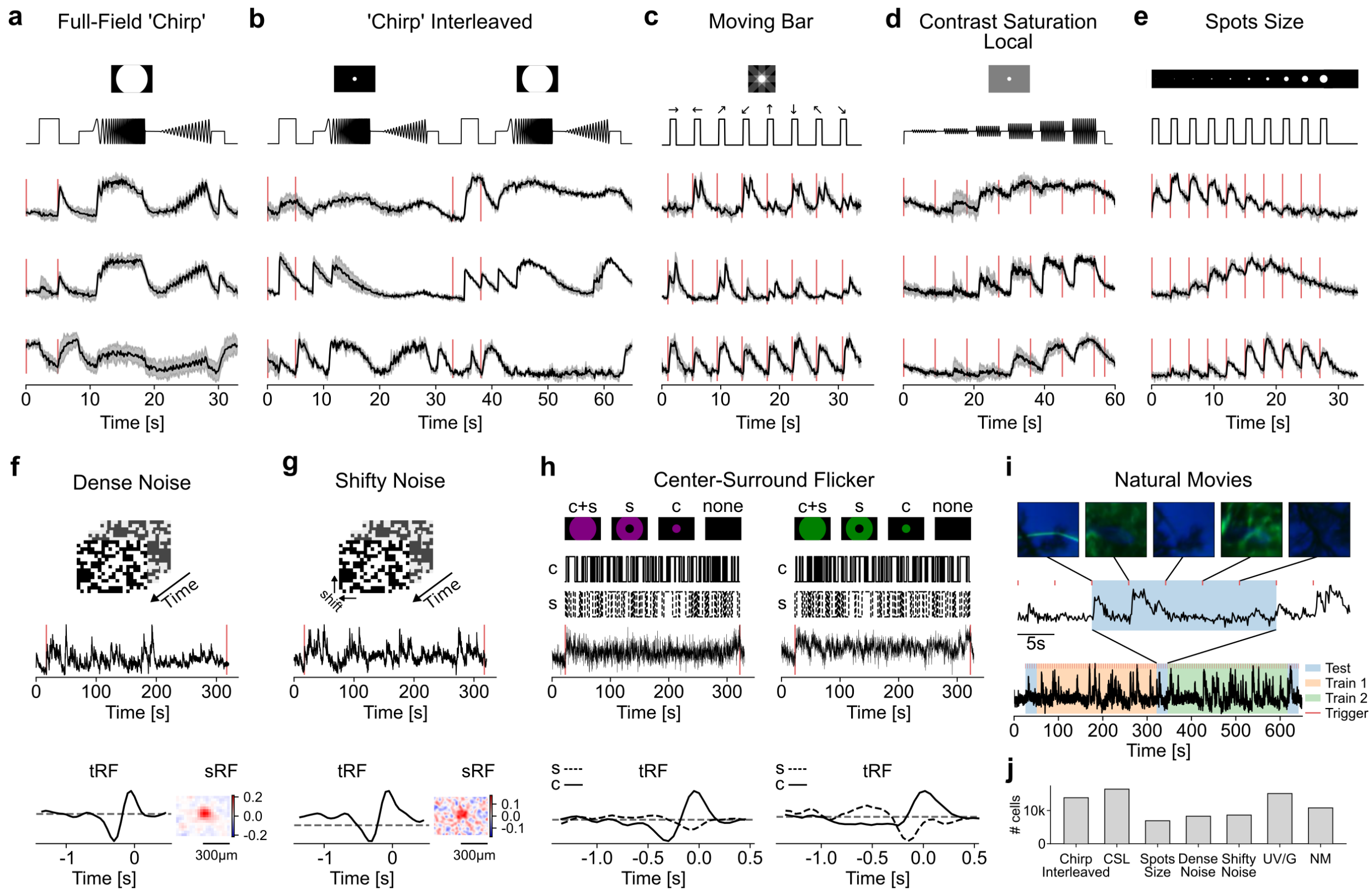

Figure S1: **Overview of visual stimulus paradigms and representative ganglion cell layer responses.** (**a**) Full-field "chirp" stimulus consisting of temporal luminance and contrast modulations presented over a large circular stimulus region. Example average responses from three representative cells are shown below. (**b-e**) Like (a) but for interleaved "chirp" stimulus alternating between a small local spot and a large full-field stimulus region (b); moving bar stimulus consisting of a bright bar moving in eight different directions, with arrows indicating bar motion direction (c); local contrast saturation (CSL) stimulus consisting of sinusoidal luminance modulations at increasing contrast levels presented within a small circular stimulus region (d); spot size stimulus consisting of bright circular spots with increasing diameters (e). (**f**) Binary dense noise stimulus used for receptive field estimation. Top: Illustration of temporally varying binary checkerboard patterns. Middle: Representative response trace. Bottom: corresponding temporal receptive field (tRF) and spatial receptive field (sRF) estimate. (**g**) Spatially shifted ("shifty") noise stimulus, where the binary checkerboard pattern was randomly shifted between frames on a fine spatial grid. Middle: Representative response trace. Bottom: Corresponding tRF and sRF estimate. (**h**) Center–surround flicker stimulus with independently modulated centre ("c") and surround ("s") regions for UV (left, magenta) and green (g; right) channels. Top: Schematic stimulus configurations. Middle: Representative response traces. Bottom: Corresponding temporal response kernels for centre and surround stimulation. (**i**) Natural movie (NM; also called mouse cam (MC) movie) stimulus. Top: Example movie frames. Middle: Expanded view of a test sequence segment. Bottom: Full recording trace with highlighted train and test segments as well as stimulus trigger times. Red vertical lines indicate stimulus trigger times. (**j**) Number of cells recorded per stimulus. Chirp was recorded for $n = 71,865$ ROIs; moving bar for $n = 79,830$.

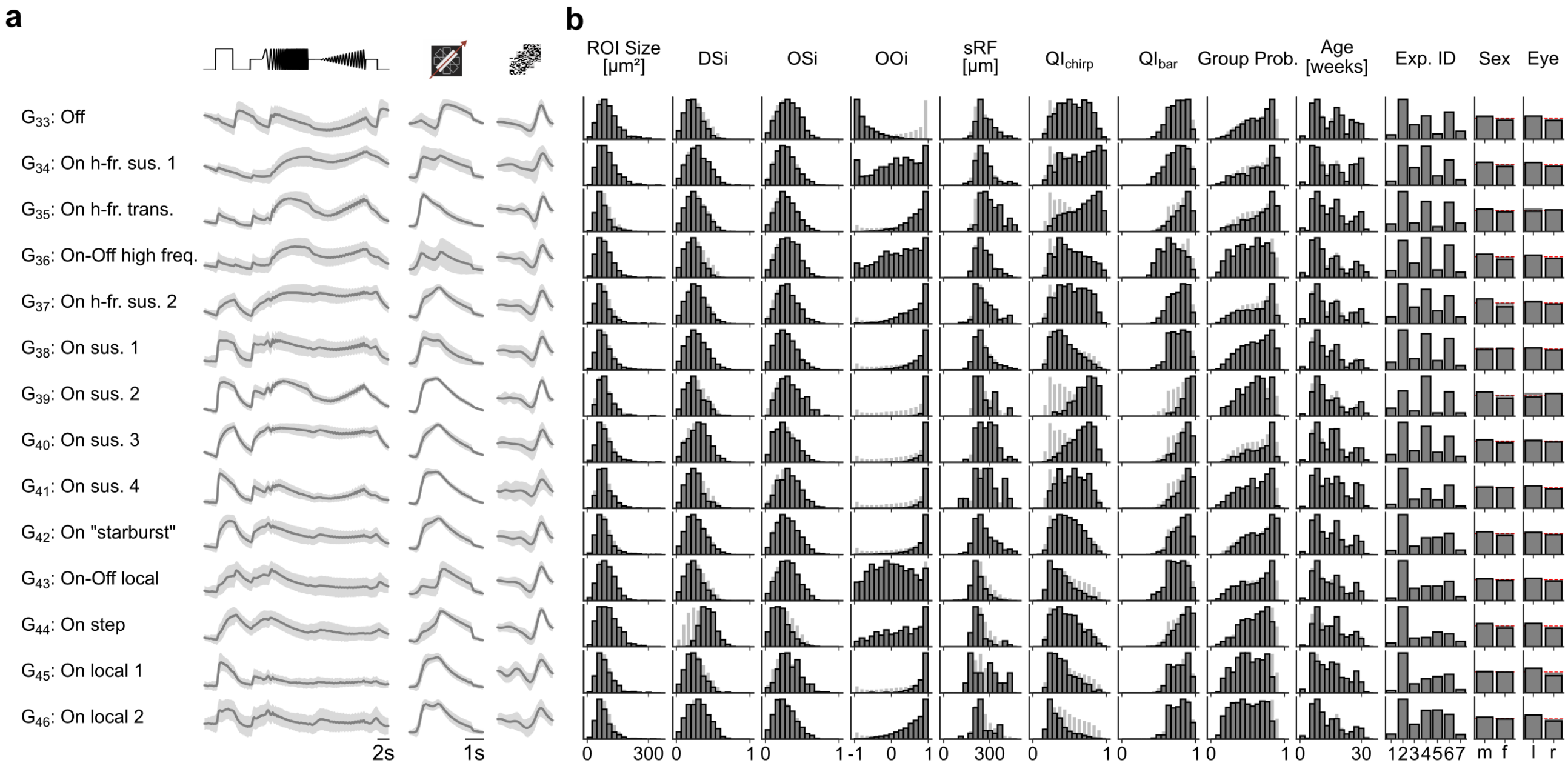

Figure S2: **Functional classification of dAC somata.** (**a**) Mean "chirp", moving bar responses, and temporal receptive fields for displaced amacrine cell (dAC) groups (G33–G46), organised by functional group. Traces represent the mean response across cells within each group, with shading indicating variability across cells. (**b**) Corresponding distributions of key physiological and experimental parameters for each dAC group, including ROI size, direction selectivity index (DSi), orientation selectivity index (OSi), On–Off index (OOi), spatial receptive field size (sRF), quality indices for "chirp" and moving bar responses, classification confidence, and associated metadata (age, experimenter ID, sex, and eye).
